# Collective bending motion of a two-dimensionally correlated bowl-stacked columnar liquid crystalline assembly under a shear force

**DOI:** 10.1126/sciadv.adg8202

**Published:** 2023-05-12

**Authors:** Yoshiaki Shoji, Ryo Komiyama, Miki Kobayashi, Atsuko Kosaka, Takashi Kajitani, Rie Haruki, Reiji Kumai, Shin-ichi Adachi, Tomofumi Tada, Naoyuki Karasawa, Hiroshi Nakano, Hisao Nakamura, Hidehiro Sakurai, Takanori Fukushima

**Affiliations:** ^1^ Laboratory for Chemistry and Life Science, Institute of Innovative Research, Tokyo Institute of Technology, 4259 Nagatsuta, Midori-ku, Yokohama 226-8503, Japan.; ^2^ Department of Chemical Science and Engineering, School of Materials and Chemical Technology, Tokyo Institute of Technology, 4259 Nagatsuta, Midori-ku, Yokohama 226-8503, Japan.; ^3^ Open Facility Development Office, Open Facility Center, Tokyo Institute of Technology, 4259 Nagatsuta, Midori-ku, Yokohama 226-8503, Japan.; ^4^ RIKEN SPring-8 Center, 1-1-1 Kouto, Sayo, Hyogo 679-5148, Japan.; ^5^ Photon Factory, Institute of Materials Structure Science, High Energy Accelerator Research Organization, 1-1 Oho, Tsukuba 305-0801, Japan.; ^6^ Kyushu University Platform of Inter/Transdisciplinary Energy Research, Kyushu University, 744 Motooka, Nishi-ku, Fukuoka 819-0395, Japan.; ^7^ National Institute of Advanced Industrial Science and Technology (AIST), Tsukuba Central 2, 1-1-1 Umezono, Tsukuba, Ibaraki 305-8568, Japan.; ^8^ Division of Applied Chemistry, Graduate School of Engineering, Osaka University, 2-1 Yamadaoka, Suita, Osaka 565-0871, Japan.; ^9^ Living Systems Materialogy (LiSM) Research Group, International Research Frontiers Initiative (IRFI), Tokyo Institute of Technology, 4259 Nagatsuta, Midori-ku, Yokohama 226-8503, Japan.

## Abstract

Stacked teacups inspired the idea that columnar assemblies of stacked bowl-shaped molecules may exhibit a unique dynamic behavior, unlike usual assemblies of planar disc– and rod-shaped molecules. On the basis of the molecular design concept for creating higher-order discotic liquid crystals, found in our group, we synthesized a sumanene derivative with octyloxycarbonyl side chains. This molecule forms an ordered hexagonal columnar mesophase, but unexpectedly, the columnar assembly is very soft, similar to sugar syrup. It displays, upon application of a shear force on solid substrates, a flexible bending motion with continuous angle variations of bowl-stacked columns while preserving the two-dimensional hexagonal order. In general, alignment control of higher-order liquid crystals is difficult to achieve due to their high viscosity. The present system that brings together higher structural order and mechanical softness will spark interest in bowl-shaped molecules as a component for developing higher-order liquid crystals with unique mechanical and stimuli-responsive properties.

## Teaser

Bowl-shaped molecules show the potential to develop liquid crystals having higher structural order and mechanical softness.

## INTRODUCTION

Since efficient synthetic methods for buckybowl molecules (i.e., subunits of C_60_) such as sumanene (*1*–*6*) and corannulene (*7*–*11*) have been established, bowl-shaped and curved π-conjugated molecules have attracted considerable attention (*12*–*14*). The major incentive for the development of nonplanar π–conjugated molecules lies in the exploration of unique properties different from those expected for planar systems. For example, bowl-shaped molecules have a dipole moment in the out-of-plane direction arising from the difference in electrostatic potentials between their concave and convex faces (*12*). They can also undergo bowl-to-bowl inversion accompanied by dipole inversion (*12*). In addition to the single-molecular properties, sumanene derivatives, in particular, are reported to form ordered one-dimensional (1D) columnar structures, similar to planar π–conjugated molecules, with convex-concave bowl stacking, providing an efficient charge-transport pathway (*15*, *16*). When bowl-to-bowl inversion is allowed in such bowl-stacked columns, a ferroelectric response could be achieved, as recently demonstrated for a columnar liquid-crystalline assembly of heterasumanene (*17*), where the dipole aligns with the bowl inversion in the direction of the applied electric field. Besides the dynamic properties arising from bowl-to-bowl inversion, it is expected that there are still uncovered behaviors, which could be derived from the characteristic shape of a bowl.

**Fig. 1.**
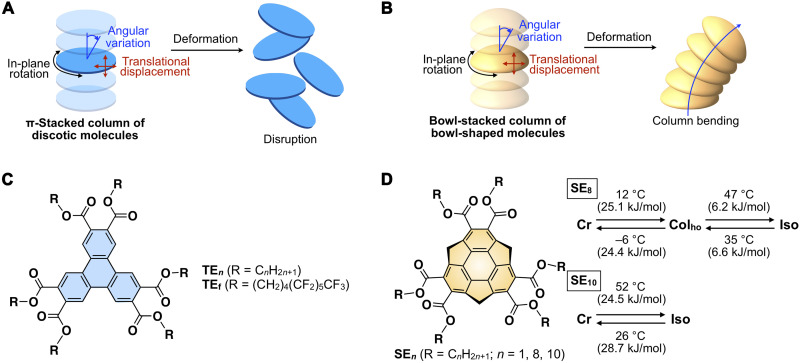
Schematic illustrations of columnar assemblies and chemical structures. (**A**) Schematic illustrations of possible movements of constituent molecules inside the columnar assemblies and the resultant columnar deformations in (A) a π-stacked discotic system and (**B**) a bowl-stacked system. (**C**) Molecular structures of triphenylene hexacarboxylic ester (**TE**) derivatives that exhibit a π-stacked, ordered hexagonal columnar (Col_ho_) mesophase (*20*, *22*), for reference. (**D**) Molecular structures of sumanene hexacarboxylic ester derivatives **SE**_***n***_ (*n* = 1, 8, and 10), and the phase-transition behavior of **SE**_**8**_ and **SE**_**10**_. Cr, Col_ho_, and Iso represent crystalline phase, ordered hexagonal columnar mesophase, and isotropic phase, respectively.

Inspired by stacked teacups in the university cafeteria, we wondered whether another mode of motion characteristic of bowl-stacked columnar assemblies, namely, bending motion, might be present. Figure 1 (A and B) shows a simple geometrical consideration of the behaviors of columnar assemblies associated with possible movements of discotic and bowl-shaped molecules. In both cases, in-plane rotation would be allowed. When considering translational displacement and angular variation, these motions should cause the disruption of columnar assemblies composed of discotic molecules (Fig. 1A). However, in the case of a bowl-stacked column (Fig. 1B), the translational displacement of the constituent molecules would be more constrained than in the discotic case, while angular variation in which the column does not collapse but bends to accommodate the molecular movement can easily occur. Accordingly, a bowl-stacked column is expected to show a flexible bending motion, which would not occur in similar columns composed of planar discotic molecules.

To test the above idea, it is reasonable to use a liquid-crystalline material that allows examination of the orientation and motion of the columns macroscopically. Such a bowl-stacked columnar LC must also uniformly align on a solid substrate over a large area, rather than one containing randomly oriented microdomains. However, hitherto known bowl-stacked columnar LCs do not show spontaneous and uniform alignment on a solid substrate (*18*, *19*). For constructing an ideal system, we took a design clue from triphenylene hexacarboxylic ester (**TE**)–based columnar LCs, which exhibit spontaneous and uniform homeotropic alignment on various solid substrates (*20*). A **TE** derivative with chiral side chains was found to form a macroscopic fluid droplet with single crystal–like structural order (*21*). Very recently, we demonstrated that a **TE** derivative (**TE**_**f**_, Fig. 1C) with semifluoroalkylated side chains not only spontaneously aligns homeotropically on solid substrates in its Col_h_ mesophase but also undergoes homeotropic-to-homogeneous alignment switching upon application of a shear force (*22*). The above mesophase behaviors have not yet been reported for extensively studied columnar LCs, e.g., those derived from hexaalkoxytriphenylenes.

In the present study, we synthesized sumanene hexacarboxylic esters **SE**_***n***_ (*n* = 1, 8, and 10; Fig. 1D) with long alkyl side chains. Depending on the length of the side chain, **SE**_***n***_ were obtained as crystalline (*n* = 1), viscose fluid-like (*n* = 8), and again crystalline (*n* = 10) materials at room temperature, and only **SE**_**8**_ exhibited a liquid-crystalline mesophase characterized by an ordered hexagonal columnar (Col_ho_) phase. Notably, in sharp contrast to usual discotic Col_ho_ LCs, **SE**_**8**_ at the mesophase temperature is very soft, like sugar syrup. Furthermore, in an LC film sample prepared on a solid substrate, the bowl-stacked columns of **SE**_**8**_ not only align homeotropically over the entire film but also undergo continuous homeotropic-to-(quasi)homogeneous alignment changes upon mechanical shearing. Encouraged by these unique properties of **SE**_**8**_, we conducted detailed experimental investigations using polarized optical microscopy (POM) and in situ x-ray diffraction (XRD) to clarify how the LC with the bowl-shaped mesogen behaves under application of a mechanical force. Through this paper, we show that bowl-shaped molecules have great potential for developing higher-order LCs with unprecedented mechanical properties and responsiveness, which are difficult to achieve using conventional rod-shaped and discotic mesogens.

## RESULTS

### Molecular dynamics calculations

**Fig. 2.**
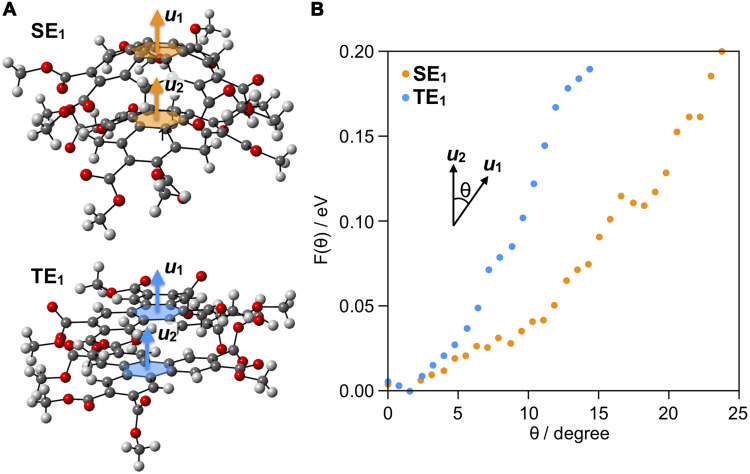
MD calculations. (**A**) Schematic illustration of stacked dimer models of **SE**_**1**_ (top) and **TE**_**1**_ (bottom). (**B**) Plots of PMF [*F*(θ)] versus inclination angle θ calculated for the stacked dimers of **SE**_**1**_ (orange) and **TE**_**1**_ (blue). The *F*(θ) minima were set to zero for both **SE**_**1**_ and **TE**_**1**_.

To gain intuitive understanding about the difference between columnar assembles of bowl-shaped and planar disc–shaped molecules (Fig. 1, A and B), we performed molecular dynamics (MD) calculations for bowl-stacked and π-stacked dimer models of **SE**_**1**_ (Fig. 1D) and **TE**_**1**_ (*20*) (Fig. 1C), respectively. We used the initial geometries taken from the crystal structures of **SE**_**1**_ (vide infra) and **TE**_**1**_ (Fig. 2A). As a measure of deformation of the dimers, we defined the angle (θ) between the two normal vectors ***u***_**1**_ and ***u***_**2**_ relative to the central six-membered rings of the upper and lower molecules, respectively (Fig. 2A). Deformability of the dimers can be evaluated using a potential of mean force (PMF). We define the probability distribution function [*P*(θ)] of finding a dimer with respect to θ, and PMF is expressed as *F*(θ) = –*k*_B_*T*log*P*(θ), where *k*_B_ and *T* are the Boltzmann constant and temperature, respectively. By means of ab initio MD (AI-MD) calculations, *P*(θ) values were calculated by sampling the atomic configurations of **SE**_**1**_ and **TE**_**1**_. Figure 2B shows plots of PMF for **SE**_**1**_ (orange) and **TE**_**1**_ (blue) with a temperature set to 400 K. For both **SE**_**1**_ and **TE**_**1**_, the most stable arrangement was found at θ *~* 0°, which corresponds to linearly stacked 1D columns. While *F*(θ) for both **SE**_**1**_ and **TE**_**1**_ can be described as a monotonically increasing function, the slope of the plots for **SE**_**1**_ is much smaller than that of **TE**_**1**_. For example, the energy value required to reach θ = 5° for **SE**_**1**_ (0.015 eV) is approximately half of that for **TE**_**1**_ (0.029 eV). The estimated energy value of thermal fluctuations at 400 K using the calculated model used is *~*10^−1^ eV, for which the θ values for **SE**_**1**_ and **TE**_**1**_ are 17° and 9°, respectively (Fig. 2B). Therefore, the bowl-stacked dimer of **SE**_**1**_ is predicted to have substantially higher deformability in terms of angular variation with respect to the stacking direction compared to the π-stacked dimer of **TE**_**1**_. We also plotted PMF with respect to the in-plane relative rotation angle and the centroid-centroid distances between stacked molecules using the same data (fig. S1). While the former (fig. S1A) is similar for **TE**_**1**_ and **SE**_**1**_, the latter (fig. S1B) shows that the distribution of distances is clearly larger and shifted toward the long-range side for **SE**_**1**_ compared to **TE**_**1**_. The picture obtained from the above simple analysis supports our intuitive prediction that bowl-stacked 1D columns may show a flexible bending motion.

### Synthesis and characterization

**Fig. 3.**
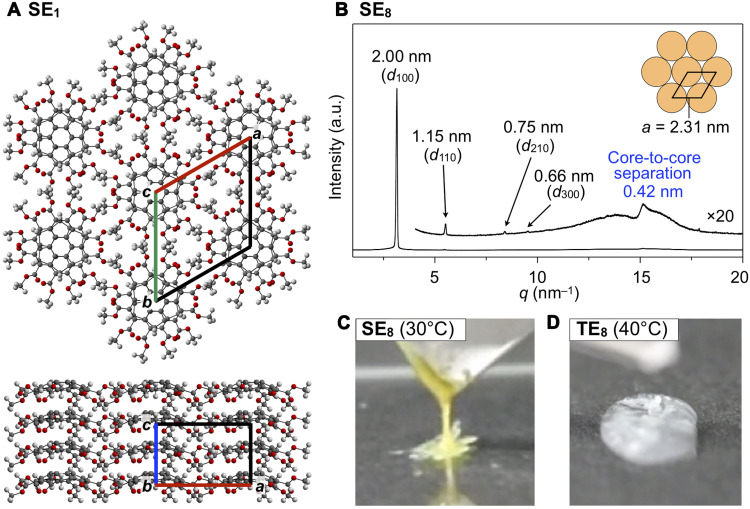
Assembly structures and appearance of sumanene derivatives. (**A**) X-ray crystal structure of **SE**_**1**_ (top; *c* axis projection, bottom; *b* axis projection). (**B**) Powder XRD pattern of a bulk sample of **SE**_**8**_ at 30°C in a glass capillary (0.5 mm in diameter) measured upon cooling from its isotropic liquid phase. Indices of the reflections are shown in parentheses. a.u., arbitrary units. (**C**) Photograph of a bulk sample of **SE**_**8**_ on a glass substrate at 30°C upon pulling up by a spatula (movie S1). (**D**) Photograph of a bulk sample of **TE**_**8**_ on a glass substrate at 40°C (Col_ho_ phase) upon scratching by a spatula, for reference (movie S2).

Methyl ester derivative **SE**_**1**_ (Fig. 1D) was synthesized by transesterification of 2,3,5,6,8,9-hexakis(phenyloxycarbonyl)sumanene (*23*) with methanol using Otera’s catalyst. Needle-shaped single crystals of **SE**_**1**_ were grown from a mixture of chloroform and hexane. Single-crystal x-ray analysis revealed that **SE**_**1**_ forms well-ordered 1D bowl-stacked columns with a staggered geometry through a convex-concave interaction (Fig. 3A, bottom). In all the columns, the convex surfaces of the bowls were aligned in the same direction in a manner similar to unsubstituted sumanene (*1*). The 1D bowl-stacked columns are laterally packed into a quasi-hexagonal structure (Fig. 3A, top).

Using procedures similar to that for **SE**_**1**_, **SE**_**10**_, and **SE**_**8**_ (Fig. 1D) were prepared. Differential scanning calorimetry (DSC) and powder XRD analysis showed that the phase diagram of **SE**_**10**_ involves only crystalline (Cr) and isotropic liquid (Iso) phases (Fig. 1D and figs. S2B and S3). In contrast, **SE**_**8**_ exhibited a mesophase between 12° and 47°C upon heating (Fig. 1D and figs. S2A and S4). The powder XRD pattern of **SE**_**8**_ at 30°C (Fig. 3B) displayed a strong peak with a *d*-spacing of 2.00 nm and weak peaks with *d*-spacings of 1.15, 0.75, and 0.66 nm due to diffractions from the (100), (110), (200), and (300) planes, respectively, of a *P*6*mm* hexagonal lattice with a lattice parameter (*a*) of 2.31 nm. A diffraction peak with a *d*-spacing of 0.42 nm, corresponding to the core-to-core separation between the bowl-stacked sumanene (*24*), was observed. Thus, the mesophase of **SE**_**8**_ is assigned to be an ordered hexagonal columnar (Col_ho_) phase. Noteworthy is the fact that, despite the formation of a highly ordered structure in the mesophase of **SE**_**8**_, the appearance and texture of its bulk sample (Fig. 3C) are very similar to those of sugar syrup (movie S1) and quite different from those of usual discotic columnar LCs. For reference, 2,3,6,7,10,11-hexakis(octyloxycarbonyl)triphenylene (*20*) (**TE**_**8**_, Fig. 1C) is a hard waxy solid in its Col_ho_ mesophase (Fig. 3D) (movie S2).

**Fig. 4.**
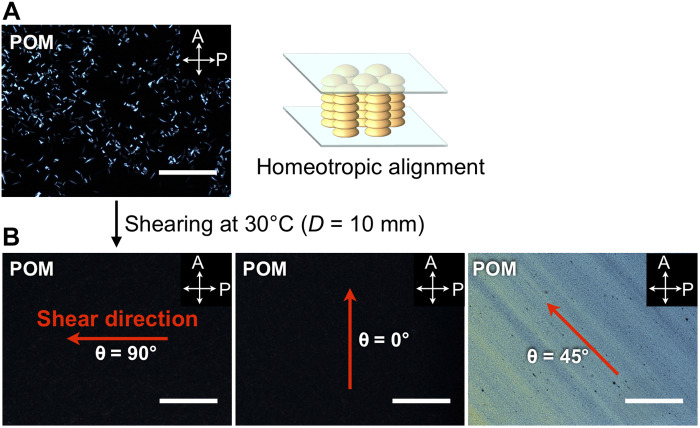
Polarized optical microscopy measurements. (**A**) POM image at 30°C of a 10-μm-thick film of **SE**_**8**_ sandwiched between two glass substrates, taken after being heated once to its melting point and then cooled to 30°C (cooling rate, 0.5°C min^−1^). Since the bowl orientation in the Col_ho_ phase cannot be determined, the drawing on the right side is just a schematic, according to the crystal structure of **SE**_**1**_, to assist the understanding of readers. (**B**) POM images at 30°C of a 10-μm-thick film of **SE**_**8**_ after mechanical shearing at 30°C (shear displacement = 10 mm). The small black spots in the right POM image are due to the glass beads (10 μm in diameter) used to adjust the film thickness to be constant. The white arrows represent the transmission axes of the polarizer (P) and analyzer (A). The red arrows and θ indicate the shear direction and angles relative to the transmission axis of the analyzer, respectively. Scale bars, 200 μm.

Figure 4A shows POM images of a film sample of **SE**_**8**_ (10 μm in thickness) sandwiched between two glass substrates. In the POM under crossed nicols, most of the areas were dark field despite the presence of some slight areas that display birefringence. The OM image showed a dendritic texture, typically observed for homeotropically aligned Col_ho_ LCs (fig. S5). Thus, **SE**_**8**_ in its Col_ho_ phase tends to adopt a homeotropic alignment on glass. Nonetheless, as is often observed for liquid-crystalline materials, when the film thickness increases, the alignment behavior deteriorated. For instance, the POM image of a 20-μm-thick film of **SE**_**8**_ (fig. S6) indicated the occurrence of randomly oriented multi-domains.

### POM measurements

Notably, when a uniaxial shear force was applied to a homeotropically aligned film of **SE**_**8**_ at 30°C, the POM image of the resultant film became completely dark when the polarizer was arranged parallel or perpendicular to the shear direction (Fig. 4B, θ = 0° or 90°). When the sample stage was rotated by 45°, a bright field appeared (Fig. 4B, θ = 45°). These observations indicate that the bowl-stacked columns of **SE**_**8**_ collectively move upon shearing, to change the alignment from homeotropic to homogeneous. In general, Col_h_ LCs with a low 1D-stacking order have low viscosity and can undergo a change in alignment upon application of a shear force, while Col_ho_ LCs feature high viscosity, and it is difficult to change the alignment using external fields. To the best of our knowledge, only two discotic columnar LCs have been reported to show such shear force–induced homeotropic-to-homogeneous alignment switching (*22*, *25*), with both examples possessing (semi)fluoroalkyl side chains. Therefore, it is obvious that the mechanical response behavior of **SE**_**8**_ is not due to an effect of the side chains but originates from the properties of the bowl-shaped mesogen.

**Fig. 5.**
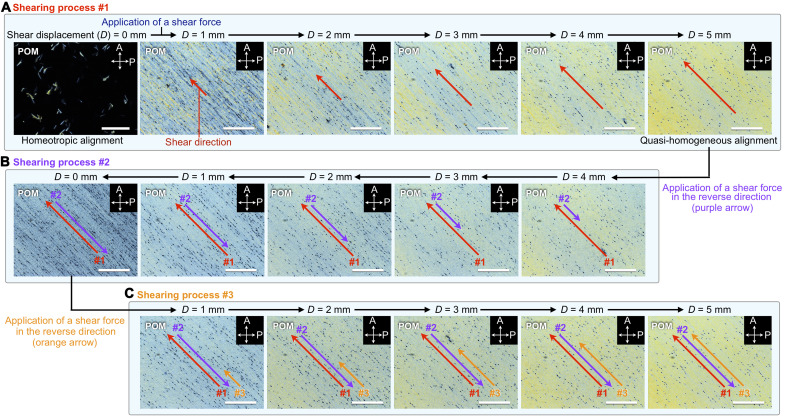
In situ POM measurements while applying a shear force. POM images of a 10-μm-thick film of **SE**_**8**_ sandwiched between two glass substrates under application of a shear force (shearing rate = 1 mm s^−1^) at 30°C, taken during (**A**) shearing process 1 (shear displacement, *D* = 0 to 5 mm), (**B**) shearing process 2 (*D* = 5 to 0 mm) in the direction reverse to that of (A), and (**C**) shearing process 3 (*D* = 0 to 5 mm) in the direction identical to that of (A). The film sample was heated once to the melting point of **SE**_**8**_ and then cooled to 30°C (cooling rate, 0.5°C min^−1^). The film sample was tilted at 45° relative to the transmission axis of the polarizer. The white arrows represent the transmission axes of the polarizer (P) and analyzer (A). The red, purple, and orange arrows indicate the shear direction. Scale bars, 200 μm. The experimental setup for the POM measurements is shown in fig. S7. The small black spots in the POM images are due to the glass beads (10 μm in diameter) used to adjust the film thickness to be constant.

For a deeper understanding of the unique behavior of **SE**_**8**_, we further investigated the shear-displacement dependence. Figure 5A shows the change in POM images at 30°C for a homeotropically aligned 10-μm-thick film of **SE**_**8**_ upon continuous shearing at a rate of 1.0 mm s^−1^ (see also fig. S7 for the experimental setup). When shear displacement (*D*) was increased (Fig. 5A, shearing process 1), the dark-field regions gradually decreased and bright-field regions appeared. The color tone in the resultant bright-field regions gradually became uniform with a decrease in the number of domain boundaries. Eventually, an almost uniform POM image due to a homogeneous alignment resulted at *D* = 5 mm. Hence, upon shearing, the direction of the longer axis of bowl-stacked columns gradually and continuously changes from perpendicular to parallel with respect to the substrate surface. This behavior is in sharp contrast to that in the Col_ho_ phase of (semi)fluoroalkylated **TE** derivative (**TE**_**f**_, Fig. 1C) (*22*), where an alignment change from homeotropic to homogeneous suddenly occurs with a threshold of shear displacement. Moreover, while the resultant homogeneous alignment of **TE**_**f**_ can be maintained for a long time as memory, this did not hold true for **SE**_**8**_. According to the POM image of a sheared film taken after being allowed to stand for 72 hours at 30°C (fig. S8), randomly oriented multidomains appear to relieve structural strains caused by the alignment change. We presume that the structural relaxation is likely due to the mechanical flexibility (i.e., large degree of freedom of molecular movement) of **SE**_**8**_ in the Col_ho_ and a surface anchoring effect that could operate at the material and substrate interface.

When a shear force was applied in the reverse direction to the homogenously aligned film of **SE**_**8**_ (Fig. 5B, shearing process 2), the overall color of the POM image was changed, with an increase in dark streaks parallel to the shear direction. However, the POM image did not return to the original dark field after the upper glass substrate was returned to the initial position (*D* = 0 mm, shear process 2). Then, a shear force was applied to the resultant sample in the same direction as shearing process 1 (Fig. 5C, shearing process 3). The change in the POM images during this shearing process is similar to those observed for shearing process 1 (Fig. 5, A and B). Last, a uniform and bright image was generated again at *D* = 5 mm. These observations indicate that the bowl-stacked columnar assembly of **SE**_**8**_ can respond flexibly and collectively to the shear force, to allow changes in alignment in the shear direction.

### In situ XRD measurements

**Fig. 6.**
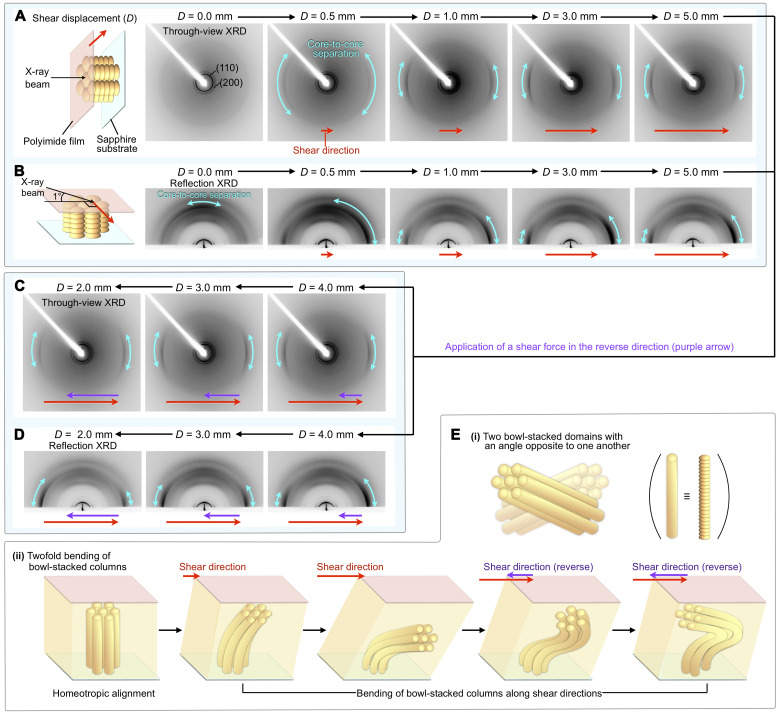
In situ XRD measurements while applying a shear force. In situ through-view (**A** and **C**) and reflection (**B** and **D**) 2D XRD images at 30°C of a 10-μm-thick film of **SE**_**8**_ sandwiched between a sapphire substrate and a polyimide sheet under application of a shear force (shearing rate = 25 μm s^−1^). The film sample was heated once to the melting point of **SE**_**8**_ and then cooled to 30°C (cooling rate, 0.5°C min^−1^). In the reflection XRD measurements, the shear direction and in-plane direction of the incident x-ray beam are perpendicular to each other. The light-blue double-headed arrows represent diffractions due to bowl-stacked sumanene. The red and purple arrows indicate the shear direction. The experimental setups for the in situ through-view and reflection XRD measurements are shown in fig. S9. (**E**) Schematic images of two possible shear force–induced behaviors of the columnar assembly of **SE**_**8**_.

To investigate the shear-force response of the hexagonal columnar assembly of **SE**_**8**_ in more detail, we performed in situ transmission (through-view) and reflection XRD measurements while applying a shear force (Fig. 6, A to D, and fig. S9). Figure 6A shows through-view XRD images of a 10-μm-thick film of homeotropically aligned **SE**_**8**_ sandwiched between a sapphire substrate and a polyimide sheet, measured at 30°C while shearing at a rate of 25 μm s^−1^. Consistent with the homeotropic alignment, before shearing (*D* = 0 mm), diffractions from the (110) and (200) planes were only observed in the small-angle region. At *D* = 0.5 mm, diffraction arcs arising from bowl-stacked sumanene (*d* = 0.42 nm) began to appear. Their width became narrower with an increase in diffraction intensity until *D* reached 1.0 mm. At a *D* value greater than 1.0 mm, no substantial change was observed in the through-view XRD images.

In situ reflection XRD measurements (incident angle of the x-ray beam = 1°; Fig. 6B and fig. S9) provided further insight into how the bowl-stacked columns of **SE**_**8**_ change the alignment. Before shearing (*D* = 0 mm), diffraction arcs due to the bowl-stacked sumanene were observed in the out-of-plane direction. Upon shearing, these gradually moved to the in-plane direction (*D* = 0.5 to 5.0 mm). The shape of the diffraction arcs did not become symmetric even at *D* = 5.0 mm, with the *d* values for the diffractions arising from the hexagonal lattice unchanged before and after shearing. Therefore, the bowl-stacked columns of **SE**_**8**_ are gradually oriented in the direction of shear force without impairing their hexagonal arrangement and eventually converge to a slightly tilted direction relative to the substrate surface. Such a quasi-homogeneous alignment of bowl-stacked columns is likely due to a surface anchoring effect, as suggested by the POM observations (Fig. 5).

Then, we applied a shear force to the resultant film at a rate of 25 μm s^−1^ in the reverse direction to the original. As shown in Fig. 6C, no detectable change was observed for the through-view XRD images at *D* values ranging from 4.0 to 2.0 mm. On the other hand, in the reflection XRD image (Fig. 6D), the inclination angle of the diffraction arcs in the in-plane direction due to bowl stacking, initially became parallel at *D* = 3.0 mm but finally inverted at *D* = 2.0 mm without moving to the out-of-plane direction. Thus, upon application of a shear force in the reverse direction, rather than regenerating a homeotropic alignment, two main homogeneous alignments with an orientation opposite to each other occur. There are two plausible scenarios that can explain the intriguing dynamic behavior of **SE**_**8**_ in response to shearing; (i) the generation of two bowl-stacked domains with inclination angles opposite to one another (Fig. 6E, i) and (ii) twofold bending of bowl-stacked columns that results in a two-orientation distribution of diffraction arcs in the in-plane direction (Fig. 6E, ii). However, the former scenario is unlikely, given the fact that no feature of domain segregation can be observed in the POM upon application of a shear force in the reverse direction (Fig. 5B). The latter scenario is reasonable when considering that the bowl-stacked column of **SE**_**8**_ can show a flexible bending motion. Although such bending of a 1D column cannot be expected for usual discotic columnar LCs, it should be possible for a 1D column composed of a bowl-shaped mesogen that has larger degree of freedom in angular variation, as predicted by the MD calculations (Fig. 2).

**Fig. 7.**
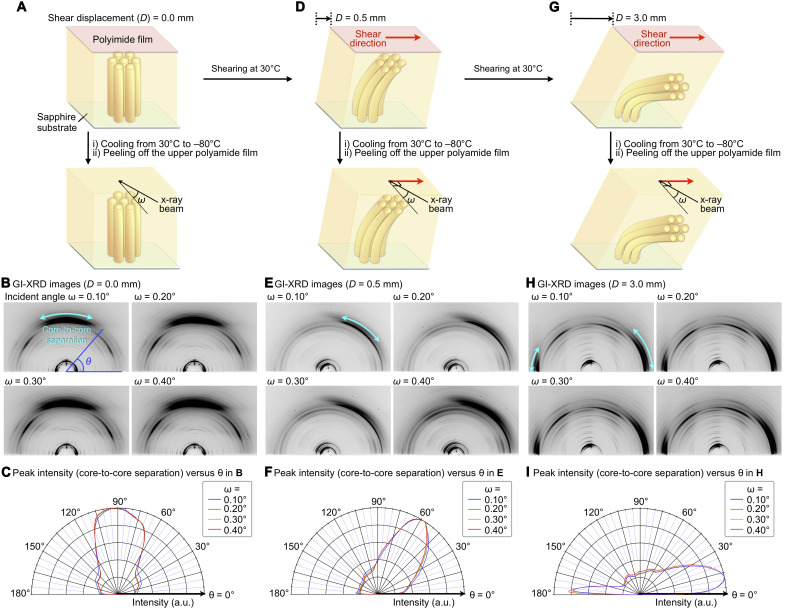
Columnar orientation along the depth direction before and after applying a shear force. GI-XRD measurements of film samples of **SE**_**8**_ with different angles of the incident x-ray beam. (**A**, **D**, and **G**) Schematic illustration of a sample preparation process. The samples were prepared by cooling from the melting point of **SE**_**8**_ to 30°C, sheared at the same temperature (shear rate = 25 μm s^−1^). The resultant film was cooled to −80°C (cooling rate, 0.5°C min^−1^), and then the upper polyimide film was peeled off. (**B**, **E**, and **H**) 2D GI-XRD images at −80°C of 10-μm-thick films of **SE**_**8**_ on a sapphire substrate prepared with a shear displacement of (B) 0.0, (E) 0.5, and (H) 3.0 mm. The shear direction and in-plane direction of the incident x-ray beam are perpendicular to each other. The light-blue double-headed arrows represent diffractions due to bowl-stacked sumanene. (**C**, **F**, and **I**) Plots of the intensity of diffractions due to bowl-stacked sumanene observed with different incident angles [0.10 (blue), 0.20 (green), 0.30 (orange), and 0.40° (red)] with respect to θ in the 2D GI-XRD images of (B), (E), and (H).

Grazing incidence XRD (GI-XRD) experiments with different angles of the incident x-ray beam provided further information on the structure developed inside the film, supporting scenario (ii) (Fig. 6E, ii). We prepared homeotropically aligned films of **SE**_**8**_ (10 μm in thickness) sandwiched between a sapphire substrate and a polyimide sheet and sheared at 30°C with displacements of *D* = 0.0, 0.5, or 3.0 mm. The resultant film was cooled to −80°C, and then the upper polyimide film was peeled off, so that the film surface can be exposed to an incident x-ray beam (Fig. 7, A, D, and G). We confirmed that this cooling process does not affect the columnar alignment inside the film (fig. S10). As expected, the 2D XRD images of a homeotropically aligned film (*D* = 0 mm) were identical, regardless of incident angle (Fig. 7, A to C). In contrast, the sheared sample (*D* = 0.5 mm) showed a clear incident-angle dependence (Fig. 7, D to F). Specifically, as the incident angle of the x-ray beam became shallower from 0.40° to 0.10°, the orientation distribution of diffraction arcs due to bowl stacking, which were tilted to the right, became larger (Fig. 7F). This indicates that the proportion of tilted bowl-stacked columns is greater near the surface. For a sheared sample (*D* = 3.0 mm) in which **SE**_**8**_ adopts a quasi-homogeneous alignment, the obtained 2D XRD images did not show any incident-angle dependence (Fig. 7, G to I). From these observations, we can conclude that, during the shearing process, the orientation distribution of the bowl-stacked columns continuously changes with respect to the depth from the film surface (Fig. 6E, ii).

**Fig. 8.**
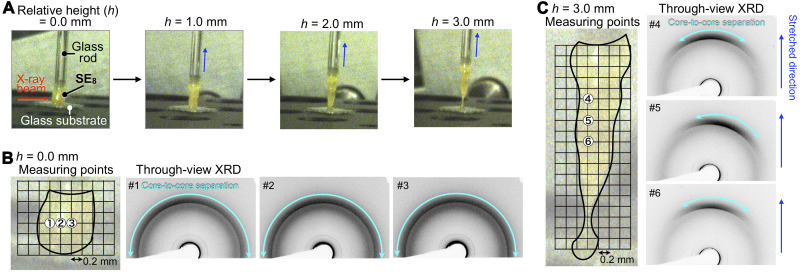
Change in the columnar alignment inside a bulk sample of SE_8_ upon stretching. (**A**) Photographs of a bulk sample of Col_ho_ **SE**_**8**_ on a glass substrate in a pulling-up process at 25°C using a glass rod with a relative height (*h*) of 0.0 to 3.0 mm. (**B** and **C**) Magnified views of the bulk sample of Col_ho_ **SE**_**8**_ and through-view XRD images collected at the measuring points shown in the magnified views (B) before (*h* = 0) and (C) after pulling-up (*h* = 3.0 mm) using the glass rod. The light-blue double-headed arrows represent diffractions due to bowl-stacked sumanene. The x-ray beam size = 80 mm by 80 μm. The experimental setup for the through-view XRD measurements is shown in fig. S11.

As already described, the Col_ho_ phase of **SE**_**8**_ is very soft and even shows a sticky nature (Fig. 3C). Figure 8A shows photographs of a bulk sample of **SE**_**8**_ on a glass substrate being touched with a glass rod and pulled up (relative heights of the glass rod; *h* = 0.0 to 3.0 mm). We were curious about how through-view XRD image of the bulk sample changes in the course of the pulling process. At *h* = 0, diffraction arcs arising from bowl-stacked sumanene were uniformly distributed, meaning that the columnar assembly does not align in any particular direction (Fig. 8B). When the LC sample was pulled up to *h* = 3.0 mm, the diffraction arcs observed at three different positions became highly oriented along the stretched direction (Fig. 8C). Thus, the bowl-stacked columnar assembly of **SE**_**8**_ can flexibly change its orientation after an external force is applied. Such orientation flexibility is often observed for lyotropic LCs and polymeric materials but is quite unique for single-component LCs composed of discrete molecules (*26*).

## DISCUSSION

On the basis our previous findings (*20*–*22*), we designed a liquid-crystalline sumanene derivative (**SE**_**8**_) to which alkyl side chains are attached via an ester linkage. We found that **SE**_**8**_ shows unique softness and remarkable dynamic behavior, which arises from bowl-stacking of the sumanene core in its ordered hexagonal columnar (Col_ho_) phase. What is particularly interesting is that, following application of a shear force, the 1D bowl-stacked columns of **SE**_**8**_ undergo a collective deformation while preserving its hexagonal 2D arrangement. In situ POM and XRD observations using specially designed experimental setups allowed us to capture the movements of **SE**_**8**_ in the Col_ho_ phase.

LCs that have an orientational order of their constituent molecules and alignment-switching ability have attracted much attention in terms of soft matter science and various practical applications (*27*–*31*). However, in LCs, there is an essential trade-off between the dimensionality of structural order and the processability. For instance, nematic LCs with low viscosity and high fluidity can easily be unidirectionally aligned by applying external stimuli including electric fields, magnetic fields, and mechanical shearing (*32*–*37*), whereas the degree of orientation of nematic LCs is generally low (*38*). In contrast, higher-order LCs such as smectic LCs and discotic hexagonal columnar (Col_h_) LCs, in which their constituent molecules assemble with 2D structural correlation, have the advantage of being able to use the intrinsic properties arising from anisotropic molecular alignments (*39*–*43*). However, alignment control and switching are difficult due to their high viscosity, which is close to crystalline in nature (*44*–*52*). This study not only reveals the collective motional behavior of bowl-stacked columns but also demonstrates the utility of bowl-shaped molecules as a mesogen of LCs to achieve both higher structural order and mechanical softness. Considering also recent progress in synthetic technology on curved π-electron systems (*12*–*14*), we believe that the present finding will stimulate the discovery of unexplored mesogens and, in turn, the development of higher-order LCs that exhibit interesting physical and/or stimuli-responsive properties.

## MATERIALS AND METHODS

### Materials

Unless otherwise stated, all commercial reagents were used as received. 1-Chloro-3-hydroxy-1,1,3,3-tetrabutyldistannoxane (*53*) and 2,3,5,6,8,9-hexakis(phenyloxycarbonyl)sumanene (*23*) were prepared according to previously reported procedures. Solid substrates were purchased from Matsunami Glass Ind. Ltd. (glass and quartz), Sigma Koki Co. Ltd. (sapphire, ϕ = 2.0 cm), Nilaco Corp. (Kapton film), Teraoka Seisakusho Co. Ltd. (Kapton tape), and Mitsubishi Chemical Corp. (SUPERIO UT) and cut into a designated size before use. Column chromatography was carried out using Kanto silica Gel 60 N (particle size, 63 to 210 μm).

### Methods

Preparative size exclusion chromatography (SEC) was performed on a Japan Analytical Industry LC-9210NEXT recycling preparative high-performance liquid chromatography system, equipped with JAIGEL-1HH, JAIGEL-2HH, and JAIGEL-2.5H columns and a multiwavelength detector (MD-2010_Plus_) using CHCl_3_ as an eluent. Nuclear magnetic resonance (NMR) spectroscopy measurements were carried out on a Bruker AVANCE III HD-500 spectrometer (500 MHz for ^1^H and 125 MHz for ^13^C). Chemical shifts (δ) are expressed relative to the resonances of the residual nondeuterated solvent for ^1^H [CDCl_3_: ^1^H(δ) = 7.26 parts per million (ppm)] and ^13^C [CDCl_3_: ^13^C(δ) = 78.0 ppm]. Absolute values of the coupling constants are given in hertz (Hz), regardless of their sign. Multiplicities are abbreviated as singlet (s), doublet (d), triplet (t), and multiplet (m). Atmospheric pressure chemical ionization–time-of-flight (APCI-TOF) mass spectrometry measurements were carried out on a Bruker micrOTOF II mass spectrometer equipped with an APCI probe. Fourier transform infrared (FT-IR) spectra were recorded at 25°C on a JASCO FT/IR-6600 Fourier-transform infrared spectrometer. DSC measurements were carried out on a Mettler-Toledo DSC 1 differential scanning calorimeter, where temperature and enthalpy were calibrated with In (430 K, 3.3 J/mol) and Zn (692.7 K, 12 J/mol) standard samples in sealed Al pans. Cooling and heating profiles were recorded and analyzed using the Mettler-Toledo STAR^e^ software system. POM was performed on a Nikon Eclipse LV100POL optical polarizing microscope, equipped with a Mettler-Toledo HS1 controller attached to a HS82 hot stage. Polarized electronic absorption spectra were recorded on a JASCO V-670 UV/VIS spectrometer equipped with JASCO RSH-744 rotation sample holder and JASCO GPH-506 polarizer.

### Synthesis

#### SE_1_

Under an argon atmosphere, a toluene solution (5.0 ml) of a mixture of 2,3,5,6,8,9-hexakis(phenyloxycarbonyl)sumanene (49.2 mg, 0.05 mmol), methanol (6.0 ml, 150 mmol), and 1-chloro-3-hydroxy-1,1,3,3-tetrabutyldistannoxane (12.8 mg, 0.02 mmol) was refluxed for 72 hours. The reaction mixture was allowed to cool to room temperature, poured into water, and extracted with CH_2_Cl_2_. The organic layer was washed successively with water and brine, dried over anhydrous Na_2_SO_4_, and evaporated to dryness under reduced pressure. The residue was subjected to column chromatography on SiO_2_ (CH_2_Cl_2_), followed by recycling preparative SEC (JAIGEL 1HH/2HH) using CHCl_3_ as an eluent. Fractions containing **SE**_**1**_ were collected and evaporated to dryness under reduced pressure. The residue was recrystallized from CHCl_3_ to give **SE**_**1**_ (12 mg) as pale-yellow crystals in 40% yield. ^1^H NMR (500 MHz, CDCl_3_): δ (ppm) 4.94 (d, *J* = 20.4 Hz, 3H), 4.14 (d, *J* = 20.4 Hz, 3H), and 3.95 (s, 18H). ^13^C NMR (125 MHz, CDCl_3_): δ (ppm) 167.4, 150.0, 148.7, 129.2, 53.0, and 44.1. FT-IR (KBr): ν (cm^−1^) 2924, 1715, 1440, 1270, 1231, and 1172. APCI-TOF mass: calcd. For C_33_H_24_O_12_ [M − H]^−^: *m*/*z* = 611.118; found: 611.121. ^1^H and ^13^C NMR spectra of **SE**_**1**_ are shown in figs. S12 and S13, respectively.

#### SE_8_

By a procedure similar to that for **SE**_**1**_, **SE**_**8**_ was obtained in 72% yield (84 mg) as a pale-yellow viscous material from 2,3,5,6,8,9-hexakis(phenyloxycarbonyl)sumanene (96 mg, 0.10 mmol), 1-octanol (10 mL, 63 mmol), and 1-chloro-3-hydroxy-1,1,3,3-tetrabutyldistannoxane (61 mg, 0.11 mmol). ^1^H NMR (500 MHz, CDCl_3_): δ (ppm) 4.92 (d, *J* = 20.4 Hz, 3H), 4.33 (t, *J* = 6.8 Hz, 12H), 4.14 (d, *J* = 20.4 Hz, 3H), 1.78 to 1.72 (m, 12H), 1.43 to 1.29 (m, 60H), and 0.88 (t, *J* = 7.0 Hz, 18H). ^13^C NMR (125 MHz, CDCl_3_): δ (ppm) 166.9, 149.8, 148.5, 129. 4, 66.0, 43.9, 31.8, 29.3, 29.2, 28.7, 26.0, 22.6, and 14.1. FT-IR (KBr): ν (cm^−1^) 2955, 2925, 2855, 1720, 1467, 1401, 1385, 1340, 1298, 1264, 1225, 1178, and 1058. APCI-TOF mass: calcd. For C_75_H_108_O_12_ [M – H]^−^: *m*/*z* = 1199.776; found: 1199.778. ^1^H and ^13^C NMR spectra of **SE**_**8**_ are shown in figs. S14 and S15, respectively.

#### SE_10_

By a procedure similar to that for **SE**_**1**_, **SE**_**10**_ was obtained in 50% yield (21 mg) a pale-yellow solid from 2,3,5,6,8,9-hexakis(phenyloxycarbonyl)sumanene (30 mg, 0.03 mmol), 1-decanol (3.0 ml, 15.7 mmol) and 1-chloro-3-hydroxy-1,1,3,3-tetrabutyldistannoxane (19 mg, 0.04 mmol). ^1^H NMR (500 MHz, CDCl_3_): δ (ppm) 4.91 (d, *J* = 20.4 Hz, 3H), 4.32 (t, *J* = 6.8 Hz, 12H), 4.14 (d, *J* = 20.4 Hz, 3H), 1.79 to 1.72 (m, 12H), 1.45 to 1.27 (m, 84H), and 0.87 (t, *J* = 7.0 Hz, 18H). ^13^C NMR (125 MHz, CDCl_3_): δ (ppm) 167.0, 149.9, 148.6, 129.5, 66.1, 44.1, 32.0, 29.7 (two peaks), 29.5 (two peaks), 28.8, 26.2, 22.8, and 14.2. FT-IR (KBr): ν (cm^−1^) 2925, 2853, 1728, 1465, 1261, 1126, 1028, 843, 802, and 700. APCI-TOF mass: calcd. For C_87_H_132_O_12_ [M − H]^−^: *m*/*z* = 1367.964; found: 1367.966. ^1^H and ^13^C NMR spectra of **SE**_**10**_ are shown in figs. S16 and S17, respectively.

### Through-view XRD measurements for bulk samples

Variable-temperature 1D XRD patterns of bulk samples were measured using the BL44B2 beamline at SPring-8 (Hyogo, Japan) equipped with an imaging-plate area detector (*54*). The wavelength (λ = 1.08 Å) of the incident x-ray beam was calibrated using cerium oxide (standard reference material 674b). The sample-to-detector distance was 286.5(1) mm. Unless otherwise stated, bulk samples in a glass capillary were measured while spinning at a rate of 100 rpm.

### In situ XRD measurements under application of a shear force on a linear motorized stage

In situ XRD experiments on a linear motorized stage (x-stage; XA05A-R101, Kohzu Precision Co.) were conducted using the BL-8A and BL-8B beamlines at Photon Factory (Ibaraki, Japan). A film sample of **SE**_**8**_ sandwiched between a sapphire substrate and a polyimide sheet was placed on a Instec HTC-402 hot stage, heated once to the melting point of **SE**_**8**_, and then cooled to 30°C (cooling rate = 0.5°C min^−1^). A shear force (the range of shear distance = 0 to 5.0 mm) was applied to the film sample at 30°C (Fig. 6). The sample was exposed to an x-ray beam (λ = 1.0 Å) with an incident angle of 90° for through-view images (Fig. 6, A and C) or 1° for reflection images (Fig. 6, B and D). 2D XRD images were collected using an imaging-plate area detector.

### GI-XRD measurements for film samples

2D XRD images of a film sample of **SE**_**8**_ were measured using the BL-8A beamline at Photon Factory (Ibaraki, Japan). The film sample sandwiched between a sapphire substrate and a polyimide sheet with a 10-μm-thick spacer was heated once to the melting point of **SE**_**8**_ on an Instec HTC-402 hot stage and subsequently cooled to −80°C. The polyimide sheet was peeled off at −80°C. The obtained film was exposed to an incident x-ray beam (λ = 1.0 Å, incident angles = 0.1 to 0.4°) with an oscillation of 1.0° for each frame. The XRD images were collected using an imaging-plate area detector.

### Single-crystal x-ray analysis

Single crystals of **SE**_**1**_ were obtained as colorless plates from a mixture of CHCl_3_ and hexane. A single crystal was coated with immersion oil (type B: code 1248; Cargille Laboratories Inc.) and mounted on a micromount. Diffraction data were collected at 90 K under a cold nitrogen gas stream on a Bruker APEX2 platform–charge-coupled device x-ray diffractometer system using graphite-monochromated Mo-Kα radiation (λ = 0.71073 Å). Intensity data were collected by an ω-scan with 0.5° oscillations for each frame. Bragg spots were integrated using the ApexII program package (*55*), and the empirical absorption correction (multiscan) was applied using the SADABS program (*56*). Structure was solved by a direct method (SHELXT version 2014/4) (*57*) and refined by full-matrix least squares (SHELXL version 2014/5) (*58*). Anisotropic temperature factors were applied to all nonhydrogen atoms. Hydrogen atoms were placed at calculated positions and refined by applying riding models.

Crystal data for **SE**_**1**_ (C_33_H_24_O_12_): pale-yellow needle, 0.43 mm by 0.05 mm by 0.05 mm, trigonal, *P*3*c*1, *a* = 13.893(4) Å, *b* = 13.893(4) Å, *c* = 7.733(2) Å, *V* = 1292.7(9) Å^3^, *Z* = 2, ρ_calcd_ = 1.574 g cm^−3^, *T* = 90 K, 2θ_max_ = 50.0°, MoKα radiation, λ = 0.71073 Å, μ = 0.121 mm^−1^, 6908 reflections measured, 1520 unique reflections, 138 parameters, *R*_int_ = 0.0342, GOF = 1.081, *R*_1_ = 0.0265 [*I* > 2σ(*I*)], w*R*_2_ = 0.0696 (all data), Δρ_min,max_ = −0.160, and 0.176 e Å^−3^, CCDC-2220104. These data can be obtained free of charge from the Cambridge Crystallographic Data Centre via www.ccdc.cam.ac.uk/data_request/cif.

### Computational details

AI-MD calculations for stacked dimer models of **SE**_**1**_ and **TE**_**1**_ were performed using the SIESTA program package (*59*). Initial geometries were used from each single-crystal structure. For the exchange correlation (XC) functional of density functional theory, van der Waals (vdW) correction functional, vdW-DF2 (*60*), and the single zeta plus polarization function–level basis set were used. To control temperature, Nose-Hoover thermostat was applied for MD. The validity of the adopted XC and basis set was confirmed by the fact that the calculation level successfully reproduced the lattice constants of the single crystal structure of **SE**_**1**_ (experimental: *a* = *b* = 13.89 Å and *c* = 7.73 Å and calculation: *a* = *b* = 13.82 Å and *c* = 7.56 Å) and the distance between the stacked molecules (experimental: *d* = 3.87 Å and calculation: *d* = 3.88 Å). To accelerate MD, mass of hydrogen atom was replaced to that of tritium. The time step of MD was taken as 0.8 fs. Trajectories were calculated for about 10 ps, where those after 2.5 ps were used for sampling. To represent flexibility of deformation of the stacked structure, inclination angle was set as collective coordinate. PMF, i.e., free-energy profiles, were calculated as a function of θ.
