# Supplementary material for: Collective bending motion of a two-dimensionally correlated bowl-stacked columnar liquid crystalline assembly under a shear force
Source: Sci Adv. 2023 May 12;9(19):eadg8202. doi: 10.1126/sciadv.adg8202 (PMC10181172; doi:10.1126/sciadv.adg8202)
Supplement: Supplementary file 1 — Figs. S1 to S17 Legends for movies S1 and S2 [file sciadv.adg8202_sm.pdf]

Science Advances
AAAS

Supplementary Materials for

Collective bending motion of a two-dimensionally correlated bowl-stacked columnar liquid crystalline assembly under a shear force

Yoshiaki Shoji *et al.*

Corresponding author: Hisao Nakamura, hs-nakamura@aist.go.jp; Takanori Fukushima, fukushima@res.titech.ac.jp

Sci. Adv. **9**, eadg8202 (2023)
DOI: 10.1126/sciadv.adg8202

The PDF file includes:

Figs. S1 to S17
Legends for movies S1 and S2

Other Supplementary Material for this manuscript includes the following:

Movies S1 and S2

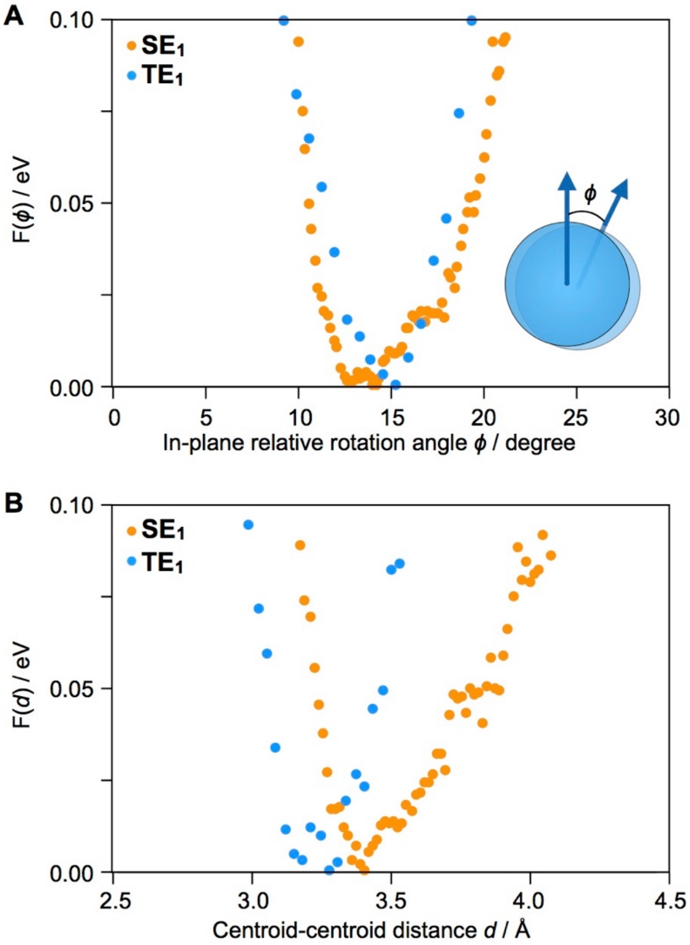

Fig. S1. Molecular dynamics calculations. Plots of PMF [$F(\phi)$ and $F(d)$] with respect to (**A**) the in-plane relative rotation angle ϕ (inset: schematic representation of ϕ between stacked molecules) and (**B**) the centroid-centroid distance d for the stacked dimers of **SE$_1$** (orange) and **TE$_1$** (blue) obtained from the same data set in Fig. 2A. The $F(\phi)$ and $F(d)$ minima were set to zero for both **SE$_1$** and **TE$_1$**.

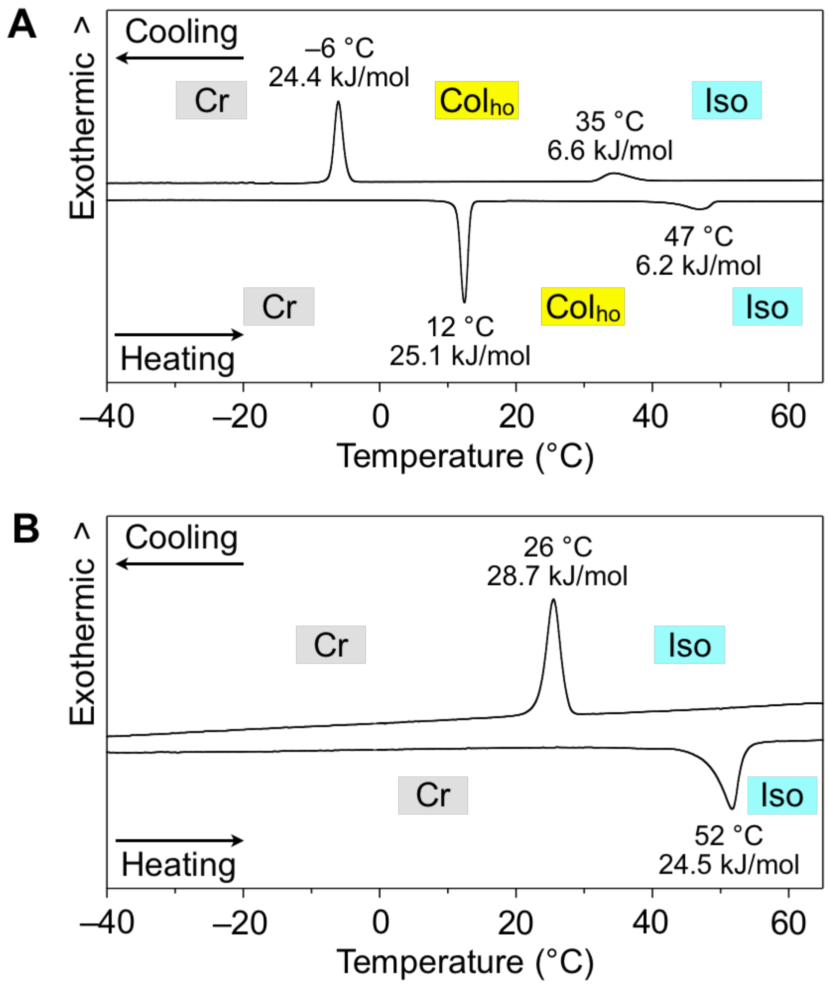

Fig. S2. Phase-transition behaviors of the sumanene derivatives. DSC profiles of (**A**) **SE$_8$** and (**B**) **SE$_{10}$** measured at a scan rate of 5.0 °C min^{-1} (second heating/cooling cycle); Cr: crystal, Col$_h$: ordered hexagonal columnar mesophase, Iso: isotropic liquid.

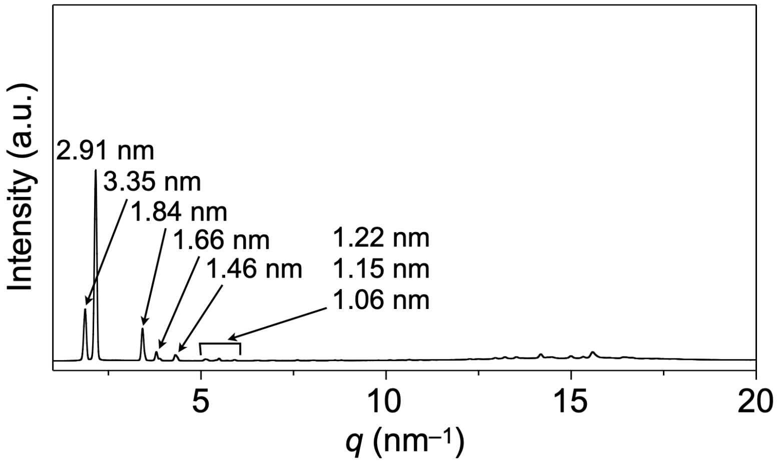

Fig. S3. Powder XRD pattern of SE_{10} in the crystalline phase. Through-view 1D XRD pattern of a bulk sample of $\mathbf{SE_{10}}$ at 25 °C in a glass capillary (0.5 mm in diameter) upon cooling from its isotropic liquid. a.u. = arbitrary units.

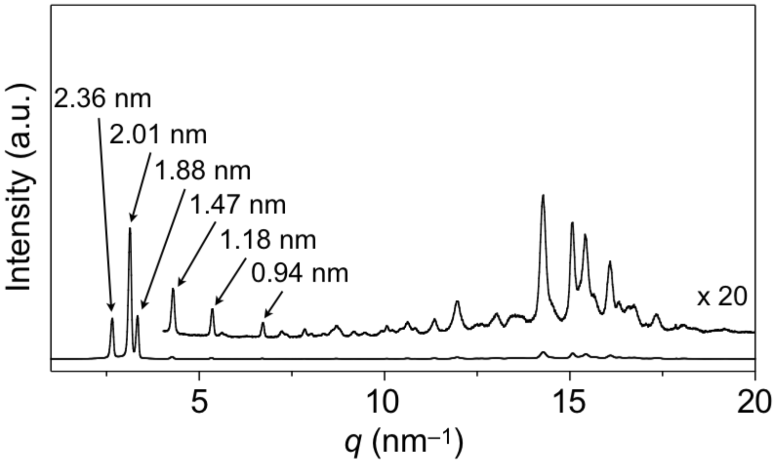

Fig. S4. Powder XRD pattern of SE_8 in the crystalline phase. Through-view 1D XRD patterns of a bulk sample of $\mathbf{SE_8}$ at –10 °C in a glass capillary (0.5 mm in diameter) upon cooling from its isotropic liquid. a.u. = arbitrary units.

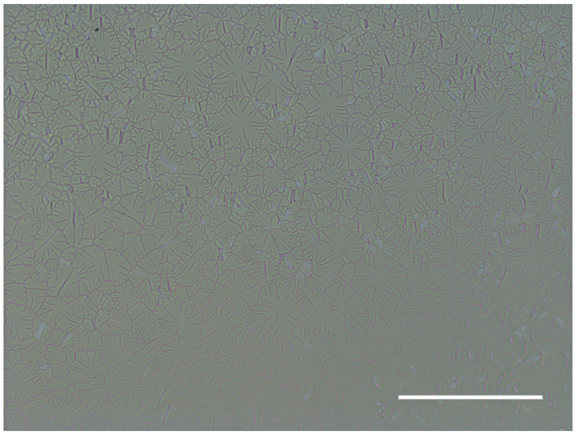

Fig. S5. Optical microscopy (OM). OM image at 30 °C of a 10 μm-thick film of **SE$_8$** sandwiched between two glass substrates, taken after being heated once to its melting point and then cooled to 30 °C (cooling rate: 0.5 °C min^{-1}). Scale bar = 200 μm.

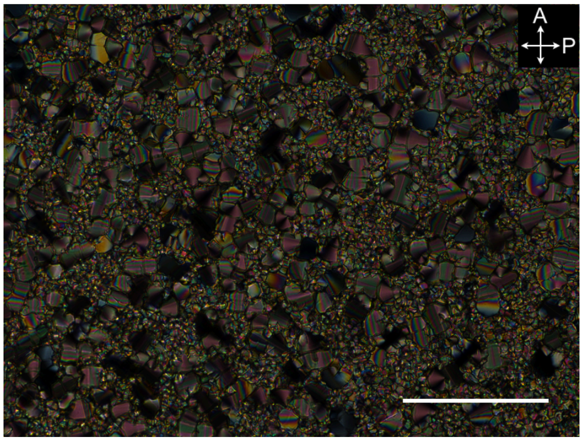

Fig. S6. Polarized optical microscopy (POM). POM image at 30 °C of a 20 μm-thick film of **SE$_8$** sandwiched between two glass substrates, taken after being heated once to its melting point and then cooled to 30 °C (cooling rate: 0.5 °C min^{-1}). The white arrows represent the transmission axes of the polarizer (P) and analyzer (A). Scale bar = 200 μm.

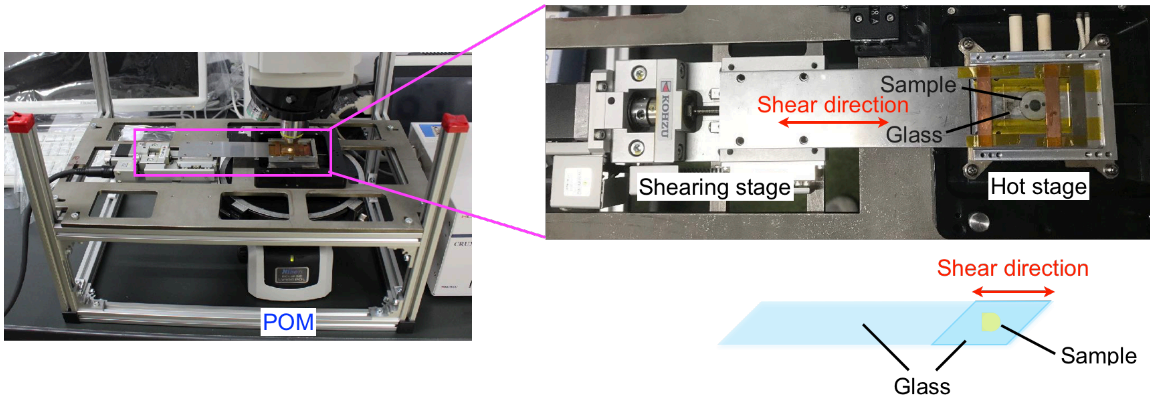

Fig. S7. Experimental setup for *in situ* POM. Photographs and schematic illustration of the experimental setup designed for *in situ* POM observations.

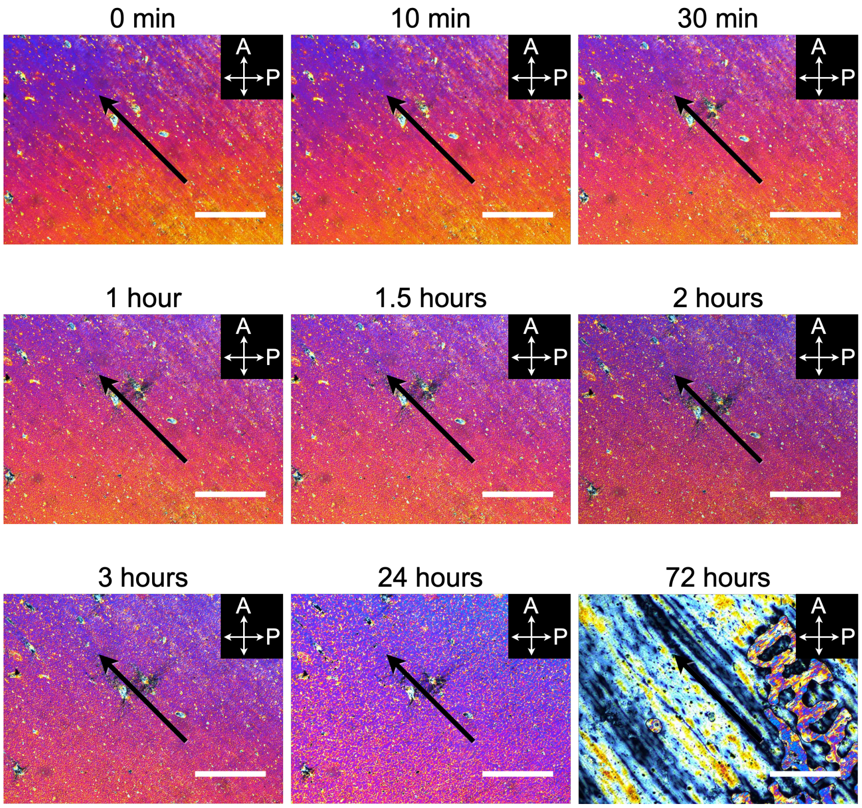

Fig. S8. Alignment behavior of SE_8 in the liquid-crystalline mesophase. POM images at 30 °C of a 10 μm-thick film of **SE_8** just after application of a shear force (top, left) and after being allowed to stand for 72 h (bottom, right). The film sample was sandwiched between two glass substrates, heated once to the melting point of **SE_8** and then cooled to 30 °C (cooling rate = 0.5 °C min^{-1}). A shear force (shear displacement = 5.0 mm) was applied at 30 °C. The white arrows represent the transmission axes of the polarizer (P) and analyzer (A), and the black arrows indicate the shear direction. Scale bars = 200 μm.

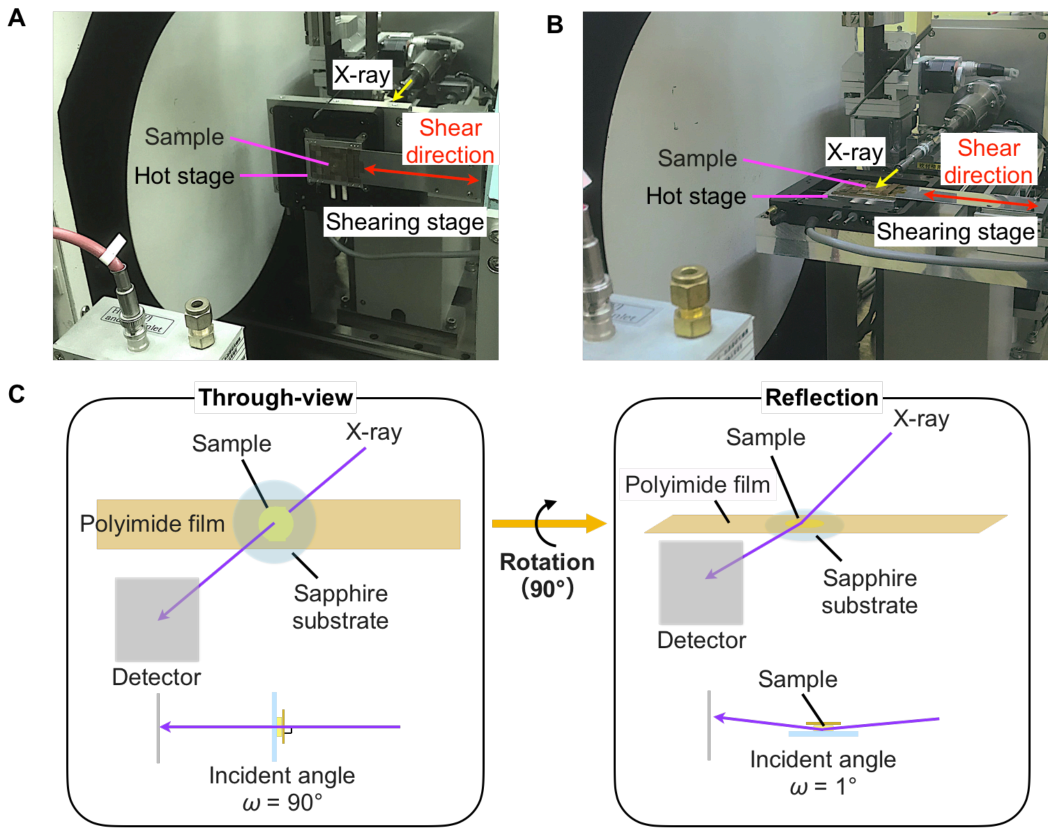

Fig. S9. Experimental setup for *in situ* XRD. (**A** and **B**) Photographs and (**C**) schematic illustrations of the experimental setup designed for *in situ* through-view (left, incident angle = 90°) and reflection (right, incident angle = 1°) XRD measurements.

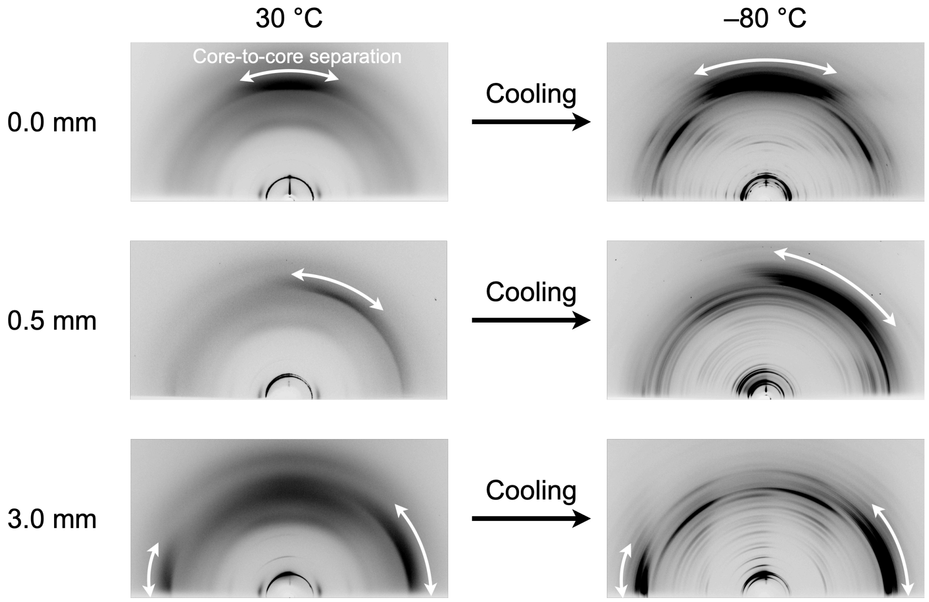

Fig. S10. Grazing incidence x-ray diffraction (GI-XRD) measurements before and after application of a shear force. 2D GI-XRD images at 30 °C (left) and –80 °C (right) of 10 μm-thick films of **SE8** on a sapphire substrate, measured after removal of the upper polyimide sheet. The samples were prepared by cooling from the melting point of **SE8** to 30 °C, sheared at the same temperature (shear displacement = 0.0, 0.5, and 3.0 mm, shear rate = 25 μm sec^{-1}), and then cooled to –80 °C. Cooling rate = 0.5 °C min^{-1}. The incident angle of the X-ray beam was 0.4°. The shear direction and in-plane direction of the incident X-ray beam are perpendicular to each other.

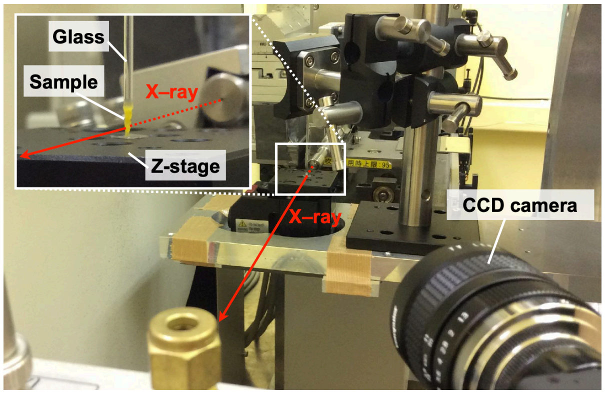

Fig. S11. Experimental setup for *in situ* XRD. Photograph of the experimental setup designed for *in situ* through-view XRD measurements (incident angle = 90°) upon pulling up a bulk sample of **SE8**.

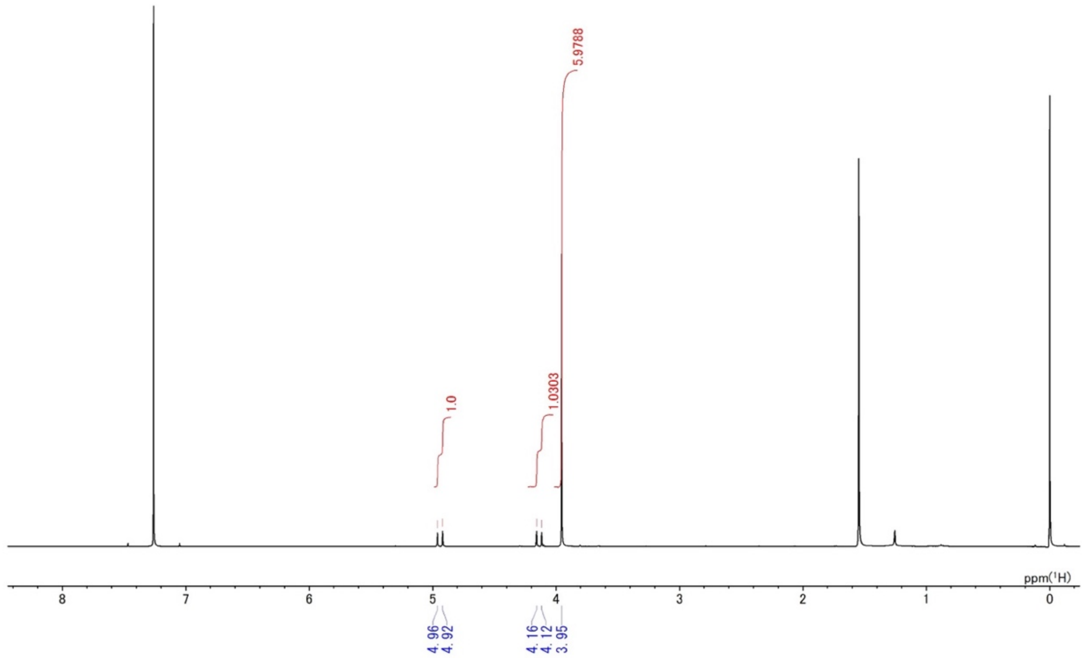

Fig. S12. **Characterization of SE$_1$.** ^{1}H nuclear magnetic resonance (NMR) spectrum (500 MHz) of **SE$_1$** in $CDCl_3$ at 25 °C.

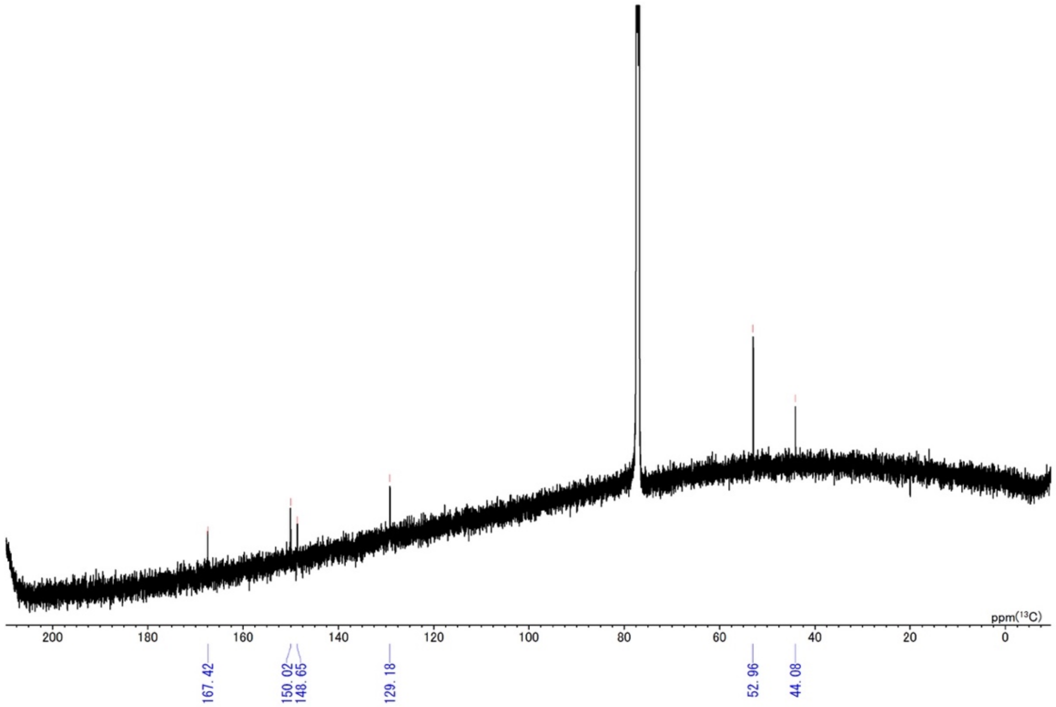

Fig.S13. **Characterization of SE$_1$.** ^{13}C NMR spectrum (125 MHz) of **SE$_1$** in $CDCl_3$ at 25 °C.

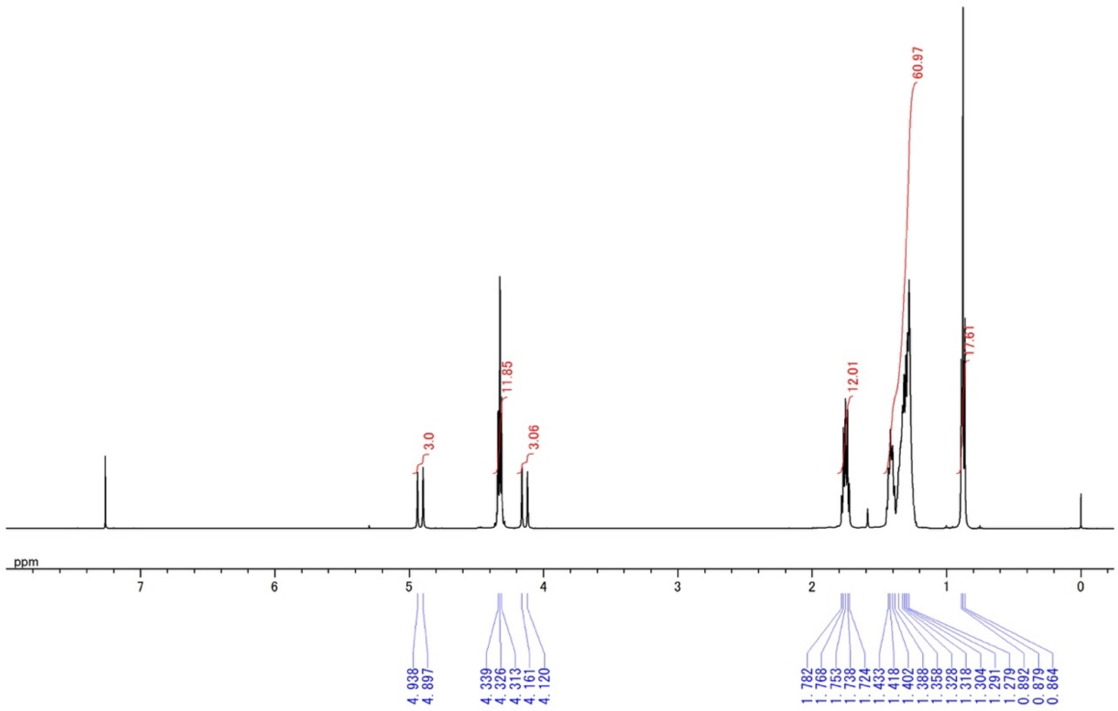

Fig. S14. **Characterization of SE₈.** ^{1}H NMR spectrum (500 MHz) of **SE₈** in $CDCl_3$ at 25 °C.

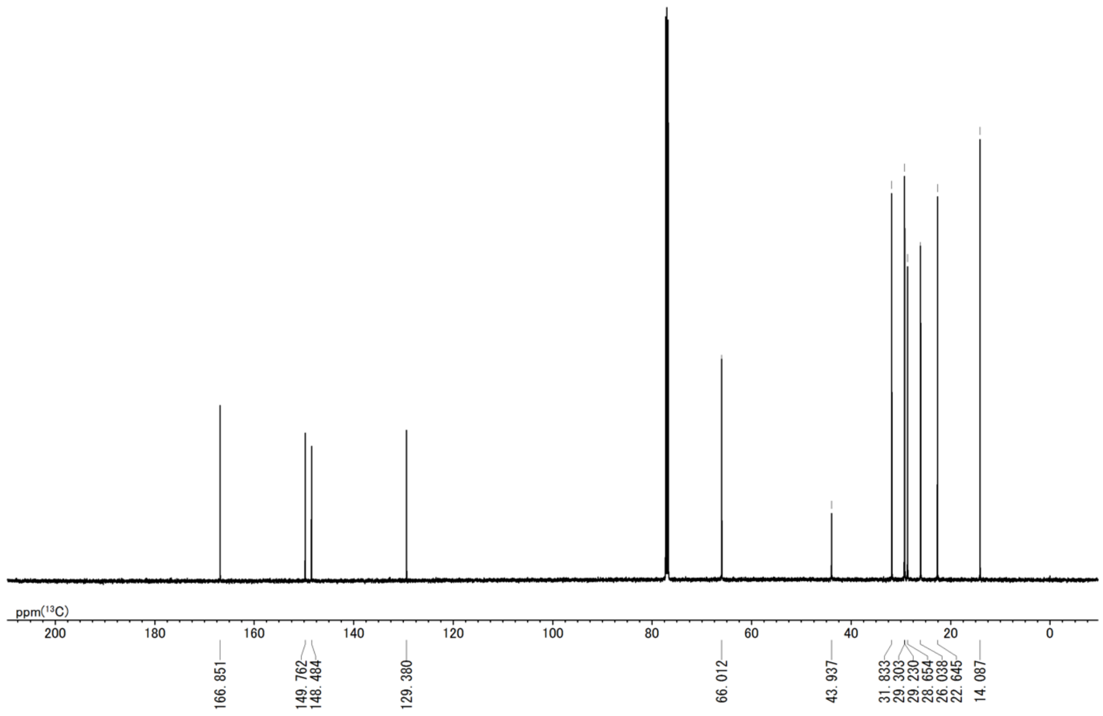

Fig. S15. **Characterization of SE₈.** ^{13}C NMR spectrum (125 MHz) of **SE₈** in $CDCl_3$ at 25 °C.

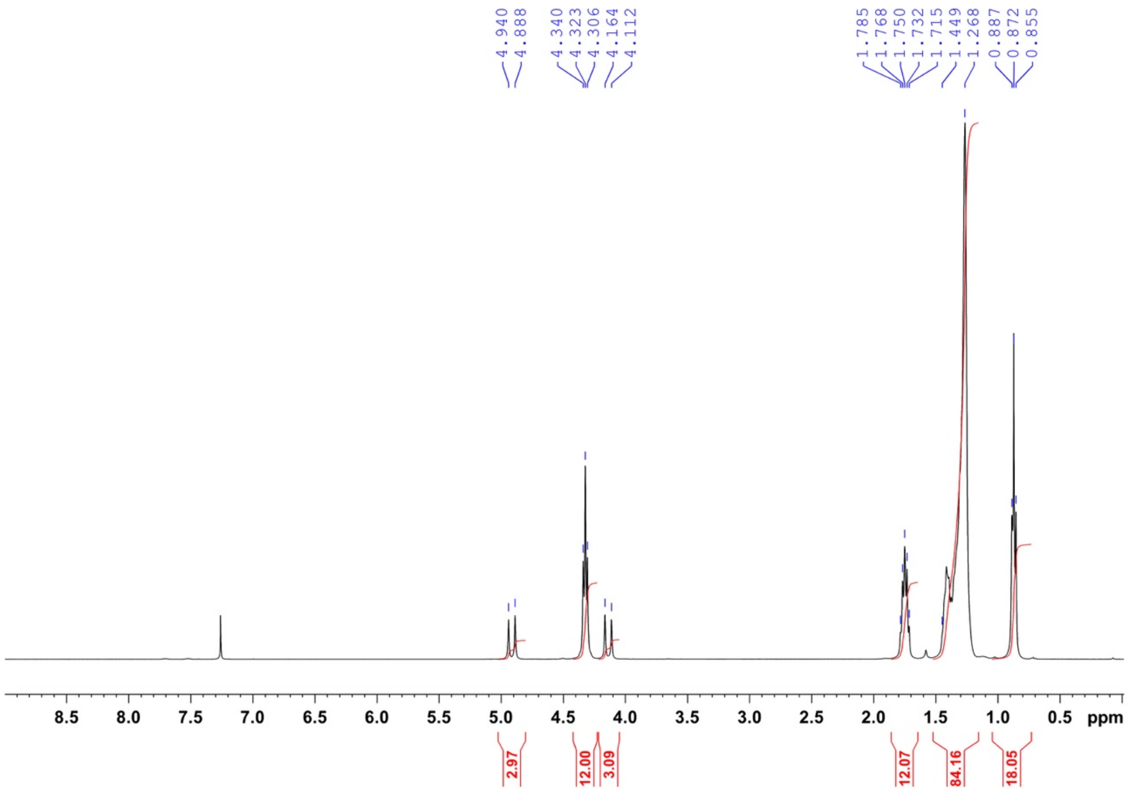

Fig. S16. **Characterization of SE$_{10}$.** ^{1}H NMR spectrum (500 MHz) of **SE$_{10}$** in $CDCl_3$ at 25 °C.

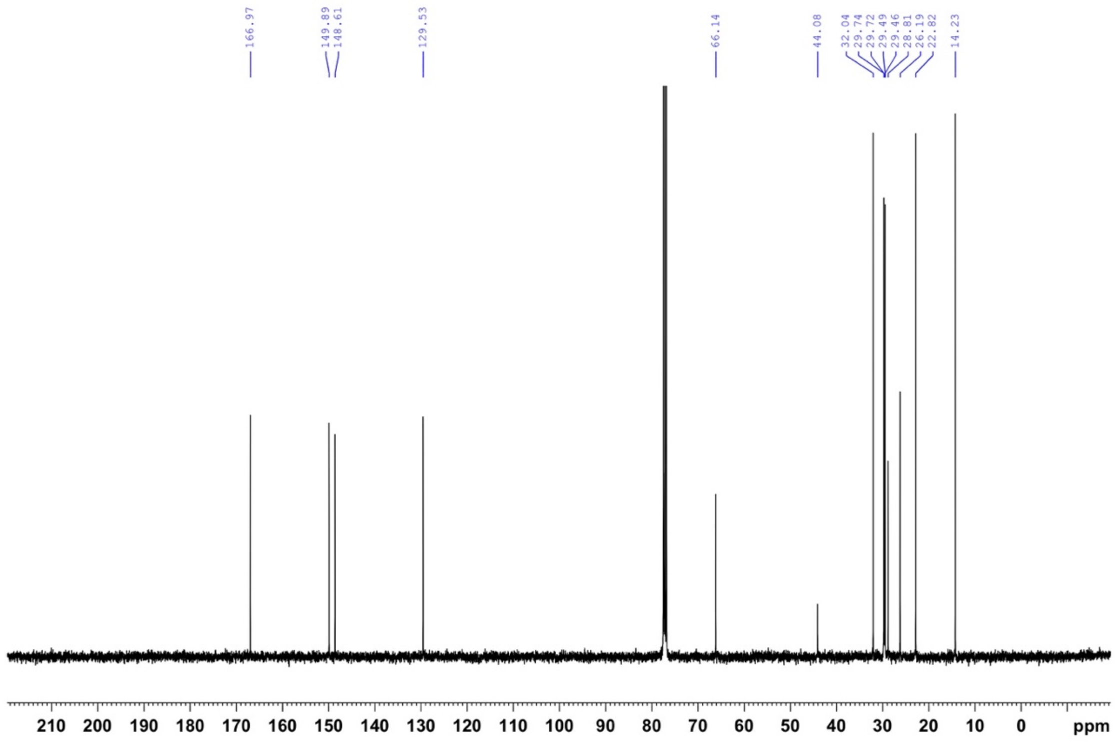

Fig. S17. **Characterization of SE$_{10}$.** ^{13}C NMR spectrum (125 MHz) of **SE$_{10}$** in $CDCl_3$ at 25 °C.

Legend of movie S1.

Movie of a bulk sample of **SE_8** on a glass substrate at 30 °C upon pulling up by a spatula.

Legend of movie S2.

Movie of a bulk sample of **TE_8** on a glass substrate at 40 °C upon scratching by a spatula.
